# Verification of the utility of the gonadotropin starting dose calculator in progestin‐primed ovarian stimulation: A comparison of empirical and calculated controlled ovarian stimulation

**DOI:** 10.1002/rmb2.12586

**Published:** 2024-05-31

**Authors:** Masato Kobanawa, Jouji Yoshida

**Affiliations:** ^1^ Kobanawa Clinic Omitama‐shi Japan; ^2^ Tsukuba ART Clinic Tsukuba‐shi Japan

**Keywords:** gonadotropin, live birth, oocyte, ovarian stimulation, progestin

## Abstract

**Purpose:**

To validate the effectiveness of a gonadotropin starting dose calculator for progestin‐primed ovarian stimulation (PPOS), we conducted a study comparing the outcomes of oocyte retrieval between a group assigned gonadotropin doses via the calculator and a control group, where doses were determined by the clinician's empirical judgment.

**Methods:**

Patients underwent controlled ovarian stimulation (COS) using the PPOS method, followed by oocyte retrieval. We assessed and compared the results of COS and oocyte retrieval in both groups. Additionally, we examined the concordance rate between the number of oocytes actually retrieved and the target number of oocytes in each group.

**Results:**

The calculated group demonstrated a significantly higher number of preovulation follicles and a higher ovarian sensitivity index than the control group. Furthermore, the discrepancy between the target and actual number of oocytes retrieved was notably smaller in the calculated group. The concordance rate between the target and actual number of oocytes was significantly greater in the calculated group.

**Conclusions:**

The gonadotropin starting dose calculator proved to be effective within the PPOS protocol, offering a reliable method for predicting the approximate number of oocytes to be retrieved, irrespective of the COS protocol employed.

## Graphical abstract

We demonstrated that the gonadotropin starting dose calculator developed based on the antagonist method is also effective for the progestin‐primed ovarian stimulation protocol. Calculated controlled ovarian stimulation (COS) was considered superior to the COS of physician's rule of thumb.

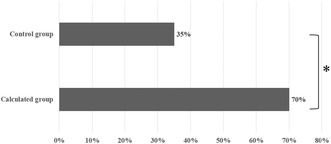

## 1 INTRODUCTION

Controlled ovarian stimulation (COS) entails the administration of gonadotropins to sustain the follicle‐stimulating hormone (FSH) level, facilitating the development of multiple follicles and the retrieval of numerous oocytes.^1^, ^2^ Preventing early surges of luteinizing hormone (LH) during COS is crucial. To achieve this, GnRH agonists, GnRH antagonists, clomiphene, and progestin have been employed.^3^, ^4^, ^5^, ^6^ Although the most effective method remains a topic of debate, GnRH antagonists and progestin‐primed ovarian stimulation (PPOS) protocols are noted for their effectiveness in preventing ovarian hyperstimulation syndrome (OHSS), a potential complication of COS.^7^ The live birth rate (LBR) in one oocyte retrieval cycle is known as the cumulative LBR^8^ and indicates COS performance, drawing attention as an important performance indicator in clinical practice.^9^

To enhance the effectiveness of COS, it is vital to retrieve the optimal number of oocytes. Therefore, identifying the appropriate initial dosage of gonadotropins for COS is essential. We created a calculator designed to enable physicians of varying levels of experience to determine a consistent starting dose of gonadotropins.^10^ However, this tool is based on data from patients undergoing the GnRH antagonist protocol, and its applicability to other COS protocols remains unknown.

To assess the utility of our calculator within the PPOS protocol, we compared the success rates in achieving the targeted number of oocytes at a clinic different from the one where the calculator was developed. Specifically, we looked at the results of COS, where the starting dose was set empirically by the physician versus when it was determined using our calculator.

## 2 MATERIALS AND METHODS

In the calculated group, we examined a cohort of 50 individuals with 50 cycles, with an average age of 35.28 ± 4.30 years, who underwent in vitro fertilization (IVF) between April 2022 and December 2022. This group underwent COS using the PPOS protocol. We employed a uniform protocol using rFSH (Gonal F; Merck BioPharma, Tokyo, Japan). At the onset of COS, the target number of oocytes to be retrieved was determined based on each patient's age, antral follicle count (AFC), and previous COS outcomes. Additionally, the duration of stimulation was set to days, and the starting dose was determined.^10^ Patients were administered a starting dose of recombinant FSH and 10 mg of medroxyprogesterone acetate (MPA) daily from the first, second, or third day of the menstrual cycle, with oocyte retrieval scheduled according to the initial plan set by the calculator.

In the control group, comprising 52 individuals with 52 cycles with an average age of 35.69 ± 4.05 years, participants also underwent IVF during the same period (April to December 2022). We also applied patients who underwent PPOS protocol in the control group. At the beginning of COS, clinicians aimed to retrieve as many oocytes as possible for the amount of AFC, but they aimed to avoid the incidence of OHSS by limiting the number of oocytes retrieved to a maximum of approximately 15. The starting dose of rFSH was administered at a daily dose of 150, 225 or 300 IU by clinician's empirical judgment for the first 6 days. Subsequently, the dose could be increased or decreased 75 IU according to the individual response during stimulation, with 300 IU as the maximum daily dose allowed. The timing for oocyte retrieval was flexible, accommodating any duration of stimulation.

When the primary follicle reached 18–20 mm in diameter, both gonadotropins and daily administration of MPA were discontinued. On the same or the following day, 250 μg of choriogonadotropin alpha (Ovitrelle/Ovidrel; Merck Biopharma, Tokyo, Japan) or 600 μg of GnRH agonist (Suprecur; CLINIGEN, Tokyo, Japan) was administered as a trigger. Oocyte retrieval occurred 34–36 h after the trigger was administered.

Following retrieval, the cumulus–oocyte complex (COC) extracted from the follicular fluid was meticulously washed and collected using a complete multi‐handling medium (FUJIFILM Irvine Scientific, California, United States). The COCs were then placed into Sequential Fert (Origio, Kanagawa, Japan) for preculture. On the day of oocyte retrieval, spermatozoa were prepared by washing them through monolayer density gradient centrifugation. The prepared spermatozoa were then selected using the swim‐up method.

For insemination, sperm was introduced to achieve a concentration of 10 × 10^4^/mL. Fertilization was verified the following morning (approximately 19 h postinsemination). During intracytoplasmic sperm injection (ICSI), spermatozoa isolated through the swim‐up method were used in 10% PVP (Irvine Scientific) at an optimal concentration. The retrieved oocytes were precultured and then denuded using a Cumulus Remover (KITAZATO Corporation, Shizuoka, Japan). ICSI was conducted after verifying spindle presence in mature oocytes using an inverted microscope (OLYMPUS IX73) equipped with a micromanipulator (NARISHIGE, Tokyo, Japan).

Following ICSI, the oocytes were cultured in Sequential Fert (Origio), and fertilization was confirmed the next morning (approximately 18 hours after ICSI).

Blood samples were collected throughout the study to evaluate levels of anti‐Müllerian hormone (AMH), FSH, LH, estradiol, and progesterone. The serum concentrations of AMH, assessed during the screening phase before the cycle's commencement, informed the initial dosage of gonadotropins. AMH levels were determined using the automated Elecsys AMH assay (Roche Diagnostics, Basel, Switzerland). Serum samples facilitated the evaluation of endocrine parameters, including FSH, LH, estradiol, and progesterone.

Outcomes from oocyte retrieval encompassed hormone levels on the day of trigger, total gonadotropins administered, and the number of preovulation follicles (>16 mm), oocytes retrieved, mature oocytes, and fertilized oocytes. Additional metrics included the follicular output rate (FORT) (the count of preovulatory follicles [16–22 mm in diameter] at the end of COS relative to the small antral follicle [3–8 mm in diameter] count at baseline),^11^ follicle‐to‐oocyte index (FOI) (the ratio between the total number of oocytes retrieved/the AFC at the start of COS),^12^ ovarian sensitivity index (OSI),^13^, ^14^ and incidence of OHSS. These outcomes were compared across both study groups. The presence of symptoms such as abdominal distension (Golan classification grade ≥1) was used to define the onset of OHSS.^15^

Mature oocytes were identified as MII oocytes that underwent ICSI and yielded zygotes with two pronuclei, as verified through insemination (conventional IVF). Fertilized oocytes were categorized as zygotes authenticated either by insemination (conventional IVF) or ICSI procedures.

This research was conducted with patients who had given informed consent. Exclusion criteria included patients with hypothalamic dysfunction, endometriosis, a history of ovarian surgery, or a history of pelvic radiotherapy and/or chemotherapy. The study evaluated the concordance rate between the actual number of retrieved oocytes and the target number of oocytes for both groups. The Mean Squared Error (MSE) and the root‐mean‐square error (RMSE) were calculated to assess the differences in the number of retrieved oocytes. Concordance was determined when the actual number of oocytes fell within the target range (10 ± 2 oocytes).

Statistical analysis used the *t*‐test, Mann–Whitney *U* test, multiple regression analysis, and chi‐square test, as applicable. A *p*‐value of <0.05 was considered statistically significant. All statistical analyses were conducted using EZR (Saitama Medical Center, Jichi Medical University, Saitama, Japan), a graphical user interface for R software (The R Foundation for Statistical Computing, Vienna, Austria) that enhances R commander with additional biostatistical functions.^16^

## 3 RESULTS

The statistical analysis methods employed for comparing patient background data were the *t*‐test and Mann–Whitney *U* test. The only parameter that showed a significant difference between the two groups was the rate of dose adjustment, with the control group exhibiting a higher rate (19.20%) than the experimental group (0.00%), as shown in Table 1.

**TABLE 1:** Patients' characteristics.

Variables	Calculated group (*n* = 50)	Control group (*n* = 52)	*p*‐Value
Age, years	35.28 ± 4.30	35.69 ± 4.05	0.62
Body weight, kg	57.71 ± 10.82	55.41 ± 10.69	0.28
AMH, ng/mL	2.84 ± 1.79	3.13 ± 2.55	0.51
AFC, follicles	11.00 ± 6.38	9.86 ± 5.04	0.32
Initial serum FSH, IU/L	9.62 ± 2.60	10.66 ± 3.01	0.06
Initial serum LH, IU/L	4.17 ± 1.50	4.63 ± 2.06	0.21
Initial serum E2, pg/mL	29.20 ± 15.48	29.15 ± 10.71	1
Administration of oral contraceptives in the previous cycle, *n* (%)	47 (94.0)	49 (94.2)	1
Duration of oral contraceptives administration, days	10.26 ± 0.90	10.63 ± 1.63	0.17
Duration of stimulation, days	10.98 ± 1.85	10.90 ± 1.72	0.83
Starting gonadotropins dose, IU	225 [100–300]	225 [150–300]	0.38
Dose adjustment, *n* (%)	0 (0.0)	10 (19.2)	0.001
Fertilization method, *n* (%)			
cIVF, *n* (%)	16 (32.0)	30 (57.7)	0.01
ICSI, *n* (%)	34 (68.0)	22 (42.3)	
Trigger method, *n* (%)			
Choriogonadotropin alpha, *n* (%)	39 (78.0)	43 (82.7)	0.62
GnRH agonist, *n* (%)	11 (22.0)	9 (17.3)	

*Note*: Data are presented as mean ± standard deviation of the mean or median [min‐Max]. Two groups were compared using a *t*‐test or Mann–Whitney *U* test, and a significant difference was judged to exist when *p* < 0.05.

Previous research has shown that factors such as age, AMH level, initial serum FSH level, and body weight have an impact on the results of oocyte retrieval.^10^ As a result, multiple regression analysis was conducted, incorporating age, AMH level, initial serum FSH level, and body weight as covariates in the multivariate analysis of oocyte retrieval outcomes. Additionally, the trigger method was included as a covariate in the analysis comparing the number of mature oocytes and the incidence of OHSS, and the fertilization method was included in the analysis comparing the number of fertilized oocytes.

Serum E2 on the trigger day was significantly higher in the calculated group (3013.87 ± 1324.08) than in the control group (2343.93 ± 1563.01) (Table 2).

**TABLE 2:** Result of COS and oocyte retrieval.

Variables	Calculated group	Control group	Univariate*p*‐value	Multivariate*p*‐value
Serum E2 on trigger day, pg/mL	3013.87 ± 1324.08	2343.93 ± 1563.01	0.02	0.04
Serum P4 on trigger day, pg/mL	1.06 ± 0.79	1.02 ± 0.91	0.83	0.9
Total gonadotropins administered, IU	2381.50 ± 782.25	2535.82 ± 759.42	0.31	0.16
Number of pre‐ovulation follicles (>16 mm), follicles	10.76 ± 4.20	8.46 ± 4.45	0.009	0.03
Number of retrieved oocytes, oocytes	11.00 ± 5.25	9.79 ± 6.43	0.3	0.63
Number of mature oocytes, oocytes	8.58 ± 4.68	6.63 ± 5.05	0.04	0.14
Number of fertilized oocytes, oocytes	7.92 ± 4.69	6.27 ± 4.84	0.08	0.17
FORT (Follicular Output Rate), %	1.28 ± 0.80	1.00 ± 0.60	0.05	0.09
FOI (Follicle‐to‐Oocyte Index), %	1.39 ± 1.30	1.10 ± 0.68	0.16	0.24
OSI (Ovarian Sensitivity Index) × 1000	4.44 [0.89–16.00]	3.07 [0.31–22.22]	0.04	–
OHSS, *n* (%)	2 (4)	2 (3.8)	1	0.82

*Note*: Data are presented as mean ± standard deviation or median [Max‐min]. Two groups were compared using a *t*‐test and multiple regression analysis. Multiple regression analysis was conducted, incorporating age, AMH levels, initial serum FSH levels, and body weight as adjustment factors for the multivariate analysis of oocyte retrieval outcomes. OSI was compared with the Mann–Whitney *U* test because it is non‐normally distributed. Additionally, the trigger method was included as an adjustment factor in the comparison of the number of mature oocytes and the incidence of OHSS. The fertilization method was also included as an adjustment factor in the analysis comparing the number of fertilized oocytes. A significant difference was determined to be present if *p* < 0.05.

The number of preovulation follicles (>16 mm) was significantly greater in the calculated group (10.76 ± 4.20) than in the control group (8.46 ± 4.45). The OSI was also significantly higher in the calculated group (4.44 [0.89–16.00]) than in the control group (3.07 [0.31–22.22]). However, no significant difference was observed in the incidence of OHSS between the two groups (Table 2).

The *t*‐test was used to compare the average discrepancy between the target and actual numbers of oocytes, whereas the chi‐square test was employed to compare the concordance rates.

**FIGURE 1:**
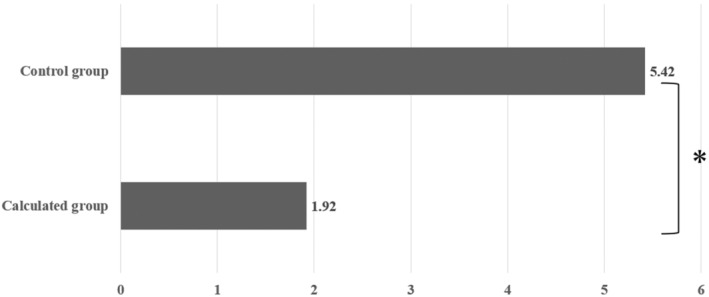
Comparison of the mean difference between the target and actual number of oocytes. The two groups were compared using the *t*‐test, with a significant difference determined to be present if *p* < 0.05. An asterisk (*) denotes a *p*‐value less than 0.05.

The mean difference between the target and actual numbers of oocytes was significantly lower in the calculated group (1.92 ± 2.87) than in the control group (5.42 ± 5.54), as indicated in Table 3 and Figure 1.

**TABLE 3:** Comparison of results between the calculated and control groups.

Outcomes	Calculated group	Control group	*p*‐Value
Mean of difference between target and actual number of oocytes, oocytes	1.92 ± 2.87	5.42 ± 5.54	0.0001
MSE (Mean Squared Error)	11.76	59.54	–
RMSE (Root‐Mean‐Squared Error)	3.43	7.72	–
Concordance rate between target and actual number of oocytes, %	70 (35/50)	35 (18/52)	0.0004

*Note*: Data are presented as mean ± standard deviation. Two groups were compared using the chi‐square test, and a significant difference was judged to exist when *p* < 0.05.

**FIGURE 2:**
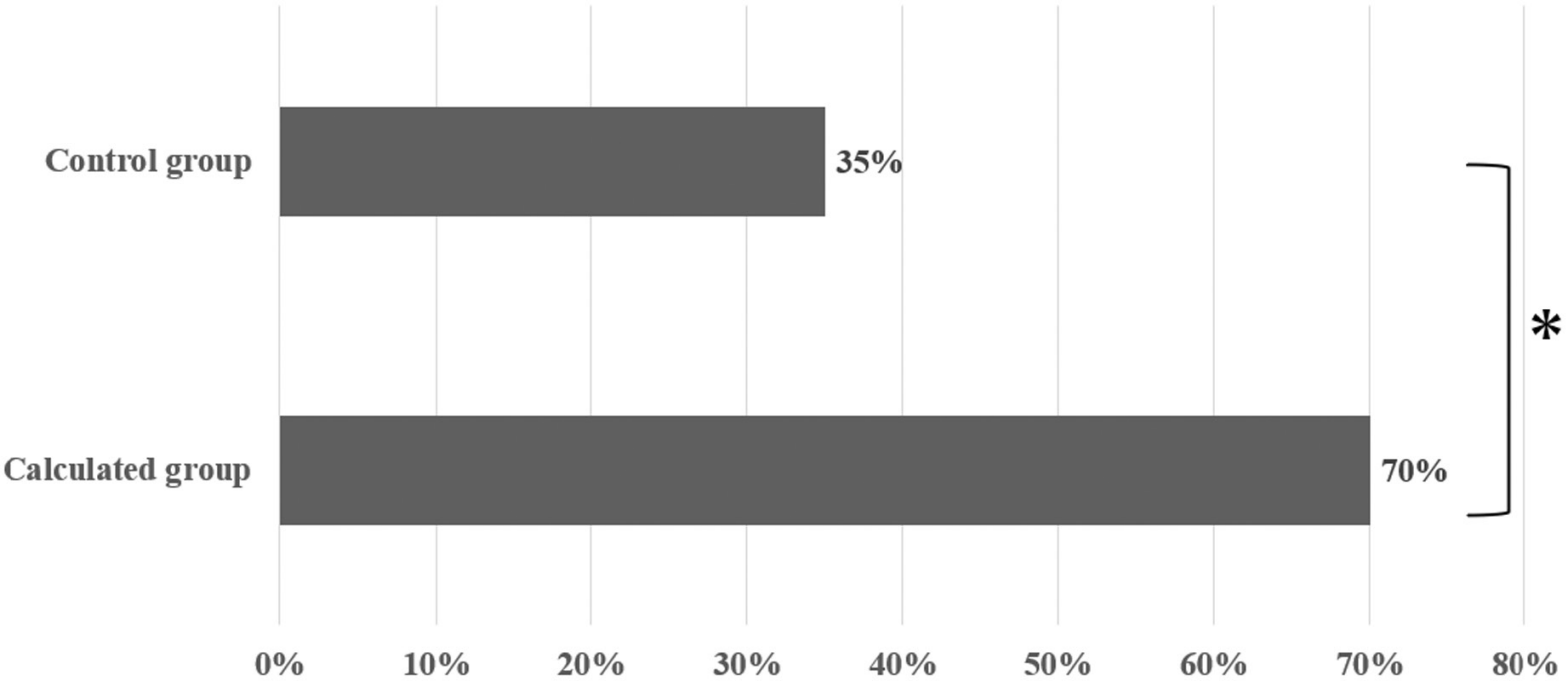
Comparison of concordance rates between target and actual number of oocytes. The two groups were compared using the chi‐square test, with a significant difference determined to be present if *p* < 0.05. An asterisk (*) denotes a *p*‐value less than 0.05.

The MSE and the RMSE were lower in the calculated group (11.76 and 3.43, respectively) than in the control group (59.54 and 7.72, respectively), as shown in Table 3. Furthermore, the concordance rate between the target and actual numbers of oocytes was significantly higher in the calculated group (70.00%) than in the control group (35.00%), as detailed in Table 3 and Figure 2.

Considering these results, the gonadotropin starting dose calculation model demonstrates a high accuracy in predicting the number of oocytes retrieved.

## 4 DISCUSSION

In this study, we investigated a calculator designed to determine the starting dose for ovarian stimulation using the antagonist method, which considers AMH values and age. This tool is potentially effective within the PPOS protocol. Developed to achieve the optimal number of oocytes retrieved, the calculator aims to mitigate risks associated with COS, such as OHSS, while enhancing the cumulative pregnancy rate and LBR.

The evidence suggests that a higher number of retrieved oocytes leads to increased availability of embryos for transfer and a higher likelihood of obtaining euploid embryos, which in turn improves cumulative pregnancy rates and LBR.^17^, ^18^, ^19^, ^20^, ^21^ However, retrieving an excessive number of oocytes can heighten the risk of OHSS^22^ and increase associated costs.^23^, ^24^

Therefore, COS should aim to retrieve an optimal number of oocytes to prevent OHSS and maximize both cumulative pregnancy rates and LBR. Studies have indicated that the number of retrieved oocytes increases with the level of FSH.^25^, ^26^, ^27^ This finding implies that lower doses of FSH can decrease the overall incidence of moderate and severe OHSS,^23^ highlighting the importance of setting the starting FSH dose in relation to the total FSH dose used in COS. Parameters, such as age, AMH, and initial FSH levels, have been identified as valuable in predicting the number of oocytes retrieved. Various parameters, including age, AMH level, AFC, FSH levels, and smoking status, have been used in previous studies to develop algorithms for determining individualized dosages of gonadotropins in COS.^28^, ^29^, ^30^, ^31^ Numerous algorithms have been reported to calculate personalized dosages of gonadotropins in COS based on these parameters.^32^, ^33^, ^34^, ^35^, ^36^, ^37^, ^38^, ^39^, ^40^, ^41^, ^42^ However, no specific algorithm has been documented for determining the individualized dosage of gonadotropins using the PPOS protocol.

The PPOS method, developed by Kuang et al.,^6^ effectively employs MPA to prevent early LH surges during COS cycles.^6^ Although the precise mechanism of progesterone's action in suppressing gonadotropin production remains unclear, animal models suggest that corpus luteum‐derived progesterone serves as a potent inhibitor of pulsatile GnRH and LH secretion.^43^, ^44^ Additionally, continuous progesterone exposure during the follicular phase may deplete LH stores of gonadotropic hormones.^45^

Currently, the PPOS protocol is widely used, demonstrating no significant difference in various key parameters compared to the antagonist method. These include the number of oocytes retrieved, fertilization rate, good quality embryo count, clinical pregnancy rate per embryo transfer, implantation rate, LBR, or early miscarriage rate compared with the antagonist method.^46^, ^47^, ^48^, ^49^, ^50^ Moreover, the PPOS protocol has also been reported to produce no significant difference in mature oocyte counts or euploid blastocysts compared with the antagonist method.^51^ Furthermore, no significant differences in the incidence of perinatal complications or neonatal prognosis were observed between the PPOS protocol and the GnRH antagonist method.^52^, ^53^

Therefore, in terms of retrieved oocyte counts, stimulation duration, and FSH levels, the PPOS protocol mirrors the antagonist method closely, yielding similar clinical outcomes. This compatibility suggests that the PPOS protocol can effectively use this calculator for dose determination.

In this study, a reproductive medicine specialist with extensive experience used this calculator, aiming to collect the number of oocytes for the AFC (FOI = 1) using a rule of thumb. Despite this, the actual number of oocytes retrieved exhibited a wide range, spanning from excessive to insufficient. The probability of achieving the initially set target number of oocytes retrieved using this calculator was 70%, significantly higher than the 35% success rate observed with decisions based on empirical rules when considering a match as the target number of oocytes retrieved ±2. This improvement can be attributed to the calculator's provision of an appropriate gonadotropin starting dose for COS. Furthermore, adherence to the predetermined duration of stimulation for oocyte retrieval also played a significant role in achieving a high concordance rate between the target and actual number of oocytes retrieved.

Stimulation duration was found to be negatively correlated with the gonadotropin dose, decreasing with higher doses and increasing with lower doses^54^, ^55^ Additionally, a Cochrane review evaluating the effect of individual gonadotropin dose selection using markers of ovarian reserve in women undergoing ART revealed that across normal responders, high responders, and poor responders, an elevated gonadotropin dose led to a reduction in the duration of stimulation.^23^ Therefore, the duration of stimulation also influenced the determination of the starting dose in COS.

In this study, we have shown that the gonadotropin starting dose calculator, originally developed for use with the antagonist method, is equally effective for the PPOS protocol. When comparing COS outcomes where the starting dose was determined by the physician's empirical judgment versus doses calculated by the tool, the latter approach was found to be superior. This was evidenced by a significantly narrower gap between the targeted and actual number of oocytes retrieved, along with lower MSE and RMSE. Moreover, the use of the calculator also tended to result in a significantly higher count of preovulation follicles and an improved FORT, indicating that COS with enhanced follicle synchronization is attainable.

As the OSI was significantly higher in the calculated group, a greater number of oocytes could be retrieved with a lower dose of gonadotropins in the calculated group than in the control group. Consequently, the use of a gonadotropin starting dose calculator for COS may prove to be cost‐effective. Although no significant difference was observed in OHSS incidence between the two groups, the higher OSI may show that the dose of gonadotropins per number of oocytes retrieved will be lower. It may be safer than the COS of physician's empirical judgment. In Japan, the PPOS protocol is widely used, especially where vitrification technology is advanced, because it offers a simpler and more affordable option for patients. The calculator developed in this study enhances COS performance within the PPOS framework and facilitates national standardization. This tool ensures that physicians, regardless of their experience level, can determine the appropriate starting dose of gonadotropins. By establishing a target number of oocytes for retrieval at each facility and calculating the individual dose needed to reach this target, unnecessary administration of FSH can be avoided. This strategy not only minimizes the risk of complications but also reduces additional costs for both patients and healthcare facilities.

The primary limitation of this study was the inclusion of urine‐derived preparations in the calculator's formulation scheme, as highlighted in a previous study.^10^ Due to the presence of protein impurities, the FSH activity in urine‐derived preparations can vary between batches. Thus, the development of a standardized protocol utilizing only rFSH would be preferable. Despite this concern, both the previous and current verifications employed a protocol exclusively based on Gonal‐f, and the alignment between the target and actual number of oocytes retrieved remained consistently high. This underscores its reliable performance without encountering significant issues.

Looking ahead, our goal is to broaden our data set and delve deeper into the suitability of this method across different COS techniques, including the GnRH agonist protocol. We are optimistic about the widespread adoption of this calculator to facilitate individualized COS approaches, ensuring the retrieval of an optimal number of oocytes regardless of the selected COS methodology.

## CONFLICT OF INTEREST STATEMENT

The authors declare no conflicts of interest.

## ETHICS STATEMENT

The protocol for the research project including human subjects has been approved by a suitably constituted Ethics Committee “Medical Corporation Kobanawa Clinic Ethic Screening Committee.”

## HUMAN RIGHTS STATEMENTS AND INFORMED CONSENT

All procedures were in accordance with the ethical standards of the responsible committee on human experimentation (facility and national) and the Helsinki Declaration of 1964 and its later amendments. Informed consent for study inclusion was obtained from all patients.

## ANIMAL STUDIES

This article does not contain any studies with animal subjects performed by any of the authors.
